# BMI1 reprogrammes histone acetylation and enhances *c-fos* pathway *via* directly binding to *Zmym3* in malignant myeloid progression

**DOI:** 10.1111/jcmm.12246

**Published:** 2014-02-27

**Authors:** Hongjie Shen, Zixing Chen, Xin Ding, Xiaofei Qi, Jiannong Cen, Yuanyuan Wang, Li Yao, Yan Chen

**Affiliations:** a Key Laboratory of Thrombosis and Hemostasis of Ministry of Health, Jiangsu Institute of Hematology, The First Affiliated Hospital of Soochow UniversitySoochow, China; b Department of Radiation Oncology, The Affiliate Hospital of XuZhou Medical CollegeXuzhou, China

**Keywords:** BMI1, myelodysplastic syndrome, chronic myeloid leukaemia, ZMYM3, acetylation

## Abstract

The polycomb group BMI1 is proved to be crucial in malignant myeloid progression. However, the underlying mechanism of the action of BMI1 in myeloid malignant progression was not well characterized. In this study, we found that the patients of both myelodysplastic syndromes and chronic myeloid leukaemia with BMI1 overexpression had a higher risk in malignant myeloid progression. *In vitro* gene transfection studies showed that BMI1 inhibited cell myeloid and erythroid differentiation induced by 12-O-tetradecanoyl phorbol-13-acetate (TPA) and histone deacetylase inhibitor sodium butyrate respectively. BMI1 also resisted apoptosis induced by arsenic trioxide. Moreover, the transcript levels of *Runx1* and *Pten* were down-regulated in *Bmi1*-transfected cells in company with histone deacetylation modification. By using chromatin immunoprecipitation (ChIP) collaborated with secondary generation sequencing and verified by ChIP-PCR, we found that BMI1 directly bound to the promoter region of *Zmym3*, which encodes a component of histone deacetylase-containing complexes. In addition, as one of the downstream target genes of this complex, *c-fos* was activated with increasing histone acetylation when ZMYM3 was suppressed in the *Bmi1-*transfected cells. These results suggested that BMI1 may reprogramme the histone acetylation profile in multiple genes through either indirect or direct binding effects which probably contributes to the malignant progression of myeloid progenitor cells.

## Introduction

Myelodysplastic syndrome (MDS) is one of the most prevalent haematological malignancies and defined as clonal bone marrow stem cell disorders with a high risk of progression into acute myeloid leukaemia (AML) [1–3]. The molecular mechanism of the MDS occurrence and progression is poorly defined. There are probably several main mechanisms contributed to MDS development: the genetic and epigenetic alterations in haematopoietic stem/progenitor cells and the changes of microenvironment including immune system [4–9]. These clonal and heritable alterations in haematopoietic stem cells of MDS patients are responsible for its tendency of malignant myeloid progression and the progenitor cells differentiation blockage as well as the dysplastic features of bone marrow hypohaemia [10]. Another most common malignant haematological disease chronic myeloid leukaemia (CML), in most of which has BCR-ABL fusion protein, also has a high risk of myeloid progression even after treatment with tyrosine kinase inhibitors [11–14]. The additional loss-of-function mutation of *Runx1* is probably one mechanism that contributes to the malignant myeloid progression [15–17]. Transformation and progression into acute leukaemia at the advanced clinical stage is the most similar clinical feature shared by MDS and CML, especially those patients with additional genomic disorders [17–19].

BMI1, the core member of polycomb repressive complex 1, is crucial for the maintenance of self-renewal capacity and undifferentiated status of stem cells [20]. The primary acting pathway of BMI1 in stem/progenitor cell is to prevent P16^INK4A/ARF^ activation by directly binding to *Ink4a/Arf* locus [21,22]. Recent studies demonstrated that BMI1 is useful in predicting MDS progression and prognosis [23]. Likewise, BMI1 is also a molecular marker for predicting prognosis of CML [24]. Although the pathogenic mechanism of MDS and CML differs from each other, they both are clonal-disordered myeloid stem/progenitor cell diseases and they both have a process of rapid transition from relative mature myelocyte to large number of myeloblast in the bone marrow at the advanced stage of the disease. Thus, some common molecular mechanism which results from different primary clonal abnormality may be shared by these two diseases to govern this dynamic process at a specific stage [25,26].

BMI1 *per. se*. does not necessarily causes leukaemogenesis, but mass data show that BMI1 is crucial in leukaemic reprogramming collaborated with other partner contributing to the development of leukaemia, such as MLL-AF9, BCR-ABL, PLZF-RARA and even *Runx1* mutation in clonal-disordered cells [27–30]. Interestingly, the N-terminal in-frame mutations (N-type) and C-terminal truncated mutations (C-type) of *Runx1* mutations exhibit two distinct molecular mechanisms: N-type of *Runx1* mutation collaborated with BMI1 overexpression leads to differentiation block and increased blastic cells, while C-type of *Runx1* mutation shows increased proliferation ability. Both of these *Runx1* abnormalities are contributed to the malignant myeloid progression [30,31]. Nevertheless, as a prognostic predictor, the BMI1 function pathway that is required for malignant myeloid progression of MDS and CML is poorly defined [23,24,28,31,32]. In the light of the potential role of BMI1 in malignant myeloid progression, we tempted to investigate the potential role of BMI1 in malignant myeloid progression and deepen the insights of its function in leukaemic pathogenesis. In our present study, we found that both MDS and CML patients with BMI1 overexpression had a higher risk in malignant myeloid progression. The *in vitro* gene transfection experiments showed that BMI1 reduced *Runx1* and *Pten* transcript levels with histone deacetylation modification. Moreover, we found that BMI1 directly bound to the promoter region of *Zmym3*, which encodes a component of histone deacetylase-containing complexes, and elevated ZMYM3 target gene *c-fos* with histone acetylation. These results suggested that BMI1 may epigenetically reprogramme the histone acetylation profile for multiple genes through either indirect or direct binding effects, which probably contributes to the malignant progression in myeloid progenitor cells.

## Designs and methods

### Patient samples

All bone marrow samples were collected from the patients in the First Affiliated Hospital of Soochow University after the approval by hospital ethical committee with written informed consents from the patients. All patients are Chinese. The MDS patients with a median age of 55 consisted of 49 newly diagnosed MDS, 40 treated MDS and eight MDS transformed AML (MDS-AML). Diagnosis of MDS was based on clinical manifestation, dysplastic bone marrow cell morphology and clonal chromosome abnormalities. Eighteen non-MDS cytopaenia patients with a median age of 52 were used as control, including iron deficiency anaemia and megaloblastic anaemia. Twenty-six CML patients in chronic phase (CML-CP) and 12 CML patients in blast phase (CML-BP) were also from the patients in the First Affiliated Hospital of Soochow University. Another matched 21 *de novo* AML (dAML), in which the percentage of bone marrow blast cells and median age were similar with the CML-BP, were chosen as control group.

### CD34^+^ cells isolation and microarray

Bone marrow mononuclear cells (BMMCs) of MDS and CML patients were separated by ficoll gradient centrifugation. CD34^+^ cell was isolated by CD34 cell isolation kit (Miltenyibiotec, Teterow, Germany). The percentage of CD34^+^ cell in sorted cell was 96.7%. The sorted cell was solved in 200 μl Trizol (Invitrogen, Carlsbad, CA, USA) and stored in liquid nitrogen. Six MDS CD34^+^ cell samples of two refractory anaemia (RA), two RA with excess blasts I (RAEB-1) and two RAEB-2 were sent to Shanghai Biochip Co. (Shanghai, China) for test by Affymetrix human genome U133 plus 2.0 array (Affymetrix, Santa Clara, CA, USA). One normal sample was used as control in array statistical analysis compared to MDS.

### Quantitative real-time PCR (Q-PCR)

Total RNA was extracted by Trizol and treated by DNase (Fermentas, Burlington, Canada) before reverse transcription. RNA was isolated from MDS CD34^+^ cells by Catrimox-14® RNA Isolation Kit (TaKaRa, Otsu, Japan). Applied Biosystems 7500 Real-Time PCR System was used to analyse the transcript expression by TaqMan probe with the Universal PCR Master Mix (Applied Biosystems, Foster City, CA, USA). Relative values of gene transcript copy number were calculated by the comparative Ct method with GAPDH as internal control. K562 total cDNA was diluted from 1 to 10^−4^ to set the standard curve. Each sample was determined in triplicate and average was calculated. Primers were designed as shown in supplemental.

### Cell culture and CFU-GM assay

Human leukaemic cell line K562 and U937 were cultured in RPMI 1640 culture medium (Invitrogen) supplemented with 10% heat-inactivated foetal bovine serum (FBS, Invitrogen). SKM-1, which was established from a patient with progression to myelomonocytic leukaemia in MDS, was cultured in IMDM culture medium (Invitrogen) supplemented with 10% heat-inactivated FBS. Cultures were incubated at 37°C in 5% CO_2_ completely humidity. For colony-forming assay, cells were cultured in the 3.5 cm diameter plate with 5*10^4^ cells/ml MethoCult®H4230 (Stem cell technologies, Vancouver, Canada) for 7 days. Then the cells were picked from the colonies and were replated for two cycles. Cluster, of which the diameter was bigger than 0.3 mm, was considered as a colony after two cycles of replating and observed under an inverted microscope CKX41 (Olympus, Tokyo, Japan).

### Production of retrovirus

The pMSCV-neo retroviral vector (MSCV) was used to construct the recombinant transfection vector containing the full-length cDNA of human *Bmi1*, which was cloned from K562 with a confirmed correct sequence. The plasmid was transfected into the PT67 package cell line (Clontech, Mountain View, CA, USA) using a 3:1 ratio of lipofectamin 2000 (Invitrogen) *versus* DNA. Six hrs after transfection, the transfection reagent was removed and replaced by IMDM (Invitrogen) with 20% FBS (Invitrogen). Cells were re-fed after 24 hrs in complete media plus 1 g/l G418 (Amresco, Solon, OH, USA). Supernatants were pooled and filtered through 0.45 μm syringe filter for immediate use. U937 and K562 were cultured in supernatant containing retroviral particles in presence of 0.6 g/l protamine sulphate (Sigma-Aldrich, St. Louis, MO, USA). Twenty-four hrs after infection, cells were moved to complete media plus 0.5 g/l G418. Subclones of *Bmi1*-transfected cell were obtained by limited dilution.

### Cell viability and differentiation induction *in vitro*

The transfected cells were cultured in media without FBS for 48 hrs and examined for cell viability by vi-cell XR cell viability analyser (Beckman Coulter, Fullerton, CA, USA). To determine the potential of induced differentiation, K562 was cultured in 20 nM TPA or 0.5 mM Sodium butyrate (Sigma-Aldrich) for 72 hrs respectively. The TPA-treated K562 cells were incubated in physiological saline for 1 hr with 0.1% nitroblue tetrazolium (NBT) and 160 nM TPA at 37°C for NBT assay. Benzidine staining assay was used to analyse the erythroid differentiation of K562 induced by 0.5 mM Sodium butyrate for 72 hrs. Cell morphology was observed under an inverted microscope CKX41.

### Western blotting assay

Samples were lysed in whole cell lysis buffer, 50 mM Tris-HCl, 150 mM NaCl, 1% SDS, 10 mM NaF by three freeze-thaw cycles followed by 30 min. on ice. Proteins were separated by electrophoresis on 10% sodium dodecyl sulphate polyacrylamide gels and transferred onto polyvinylidene fluoride membrane. After blocking with 5% dry milk/0.1% Tween-20, the membrane was incubated with primary antibody (BMI1: MAB33341, R&D Systems, Minneapolis, MN, USA) in the 1% dry milk/0.1% Tween-20 solution. The bound antibodies were detected by anti-IgG conjugated peroxydase.

### Flow cytometric analysis

Cells, washed twice with PBS, were incubated with fluorescence-conjugated antibodies (antibodies of CD15, CD71, GPA and IgG were purchased from Beckman coulter) for 30 min. at 4°C and subsequently analysed by flow cytometry (FCM) 500 (Beckman Coulter). Isotype primary conjugated antibodies served as a negative control. Annexin V (A13199; Invitrogen) was used to detect cell apoptosis induced by 5 μM arsenic trioxide (ATO; Sigma-Aldrich) for 48 hrs. Samples were prepared and analysed in duplicate, and a minimum of 5000 cells was counted for each sample.

### Chromatin immunoprecipitation assay and ChIP-seq/PCR

The chromatin immunoprecipitation (ChIP) DNA samples were prepared by ChIP Assay Kit (17-295; Upstate, Millipore, Billerica, MA, USA). Cross-linked DNA was sheared to 300–1000 bp in length by SONICS Uibra cell (Sonics & Materials, Newtown, CT, USA). Every ChIP assay contains more than 1 × 10^6^ cells. ChIP grade antibodies against BMI1 (ab14389; Abcam, Cambridge, MA, USA), acetyl-H3 (06-599; Millipore), acetyl-H4 (06-598; Millipore), acetyl-H3K27 (ab4729; Abcam), ubiquitin-H2A (05-678; Millipore), trimethyl-H3K9 (ab8898; Abcam), trimethyl-H3K27 (ab6002; Abcam) and normal Mouse IgG(12-371; Millipore) were used at 2 μg per condition. The products of ChIP were sent to Shanghai Biochip Co. for second-generation sequencing by illummina HiSeq 2000.

### Statistical analysis

The statistical analysis was evaluated with the Mann–Whitney test. Linear regression was used to evaluate the correlation of MDS international prognostic scoring system score (IPSS) and *Bmi1* transcript level in CD34^+^ cells. *P*-values less than 0.05 were considered statistically significant.

## Results

### BMI1 correlated with a high risk of malignant myeloid progression in both MDS and CML patients

**Figure 1:**
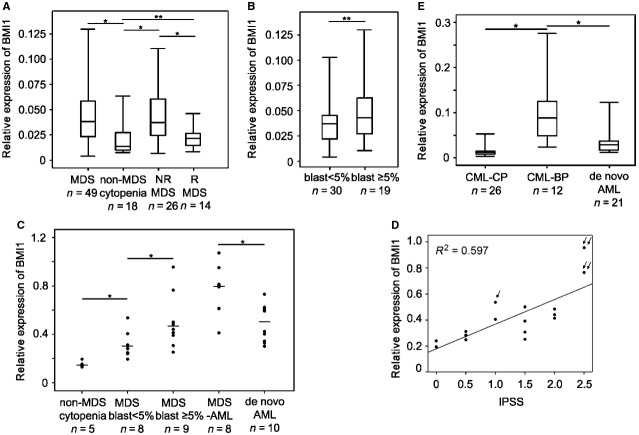
Bmi1 transcript expression in myeloid progression (A–E). The Bmi1 transcript expression levels were all performed by quantitative PCR analysis (Q-PCR). GPADH was as control. Relative expression was calculated by the comparative Ct method. (A) The relative Bmi1 transcript expression in myelodysplastic syndromes (MDS) and non-MDS cytopaenia bone marrow mononuclear cells (BMMCs). “NR MDS” means MDS not in remission. “R MDS” means MDS in remission. (B) The relative BMI1 expression in two MDS groups separated by the ratio of blast cells in MDS bone marrow smear. (C) The transcription of Bmi1 in non-MDS cytopaenia, MDS, MDS transformed acute myeloid leukaemia [MDS-acute myeloid leukaemia (AML)] and *de novo* AML (dAML) CD34+ cells. (D) The correlation of BMI1 transcription in MDS CD34+ cells and international prognostic scoring system score was evaluated by linear regression. The patient with one arrow progressed to refractory anaemia (RA) with excess blasts (RAEB) from RA and the patients with double arrows transformed to AML from RAEB. (E) The relative Bmi1 transcription in chronic myeloid leukaemia in chronic phase (CML-CP), CML in blast phase (CML-BP) and matched dAML BMMCs. (A, B, E) Quartile figure is used to present the data, and bar represents the median of the data. “*” indicates statistically significant *P* < 0.05; and “**” means without statistically significant.

Q-PCR showed that *Bmi1* transcription was up-regulated in MDS BMMCs compared to non-MDS cytopaenias. The *Bmi1* transcription in MDS BMMCs was markedly decreased after MDS patients achieved morphological and cytogenetic remission, *P* < 0.05 (Fig.1A). Further statistical analysis showed that the *Bmi1* transcription in MDS BMMCs displayed an increasing tendency from the group with blasts lower than 5% to the group with blasts higher than or equal to 5%, but a statistically significance was not reached (Fig.1B). However, a significance difference of the *Bmi1* transcription in MDS CD34^+^ cells had been found between these two groups, *P* < 0.05 (Fig.1C). In addition, the *Bmi1* transcription in MDS CD34^+^ cells also positively correlated with the IPSS, *R*^2^ = 0.597 *P* < 0.01. These results indicated that the abnormal transcript level of *Bmi1* in MDS was mainly in undifferentiated stage. Interestingly, the MDS patients with higher transcript level of *Bmi1* had a higher frequency of disease progression towards AML after diagnosis, as was demonstrated in one MDS-RA case progressed to MDS-RAEB and two MDS-RAEB cases transformed into AML in our patient cohort (Fig.1D). Furthermore, the *Bmi1* transcription of MDS-AML CD34^+^ cells was even much higher than that of dAML (Fig.1C). Similar results were found in CML myeloid progression. The *Bmi1* transcription in CML-BP BMMCs was much higher than that in CML-CP BMMCs, *P* < 0.05. Same as MDS, the *Bmi1* transcription of CML-BP was also much higher than that of matched dAML, *P* < 0.05 (Fig.1E).

### The higher level of BMI1 in leukaemic cells changed their phenotypes

**Figure 2:**
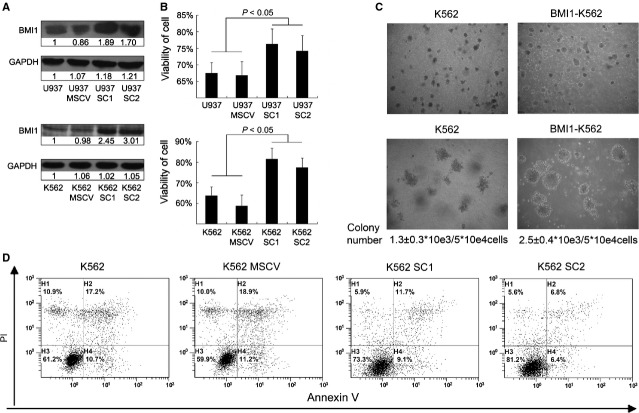
BMI1 enhances the malignancy of leukaemic cells (A) Western blotting of BMI1 in subclones of transfected U937 and K562 cells. Numbers are the folds compared to the first line control. SC1 and SC2 represent two subclones of Bmi1 transfected U937 and K562 cells. (B) The viability of cell was examined by vi-cell XR cell viability analyser after cells were cultured without foetal bovine serum for 72 hrs, *n* = 3. (C) Colony forming assay of K562 and Bmi1-transfected K562. Cells were cultured in MethoCult®H4230 with 5*104cells/mL for 7 days and then replated two cycles. Numbers are colonies with diameter larger than 0.3 mm, *n* = 3. The colony number of Bmi1-transfected K562 was greater than control with statistical significance, *P* < 0.05. The colony number means the total number of colonies/number of cell input after two cycles replating. (D) The apoptosis ratio of K562 after 48 hrs of 5 μM arsenic trioxide treatment tested by annexin V and propidium iodide in flow cytometry.

As there was no distinct MDS cell line available and the K562 cell line was established from the bone marrow of a CML patient, the open reading frame of *Bmi1* was transfected into K562 and U937 leukaemic cell by retroviral expression system [33]. Two subclones of K562 transfectant with more than 2-folds BMI1 levels and two subclones of U937 transfectant with about 2-folds BMI1 levels were obtained by limited dilution (Fig.2A). After the cells were cultured in media without FBS for 72 hrs, the viabilities of subcloned transfectant cells were higher than that of controls, *P* < 0.05 (Fig.2B). Although the overexpression of BMI1 in K562 and U937 cells seemed not to increase the cell proliferation, the colony-forming assay showed that the colony size of *Bmi1*-transfected cell colony was markedly bigger than that of control cells. After two cycles of colony-replating assay, the number of *Bmi1*-transfected cell colony, of which the diameter was larger than 0.3 mm, was 2.5 ± 0.4*10^3^/ml which was significantly greater than 1.3 ± 0.3*10^3^/ml of control cell colony, *P* < 0.05 (Fig.2C). On the other hand, the annexin V assay performed by FCM demonstrated that the *Bmi1*-transfected K562 cells became less apoptotic compared with the control cells after treatment with 5 μM ATO for 48 hrs. BMI1 seemed not to reduce the ratio of early apoptotic cells, but mainly slow down the proceeding of early apoptotic cells into late apoptotic cells, indicating that the overexpression of BMI1 allowed cells becoming more resistant to apoptosis induced by ATO in K562 (Fig.2D).

**Figure 3:**
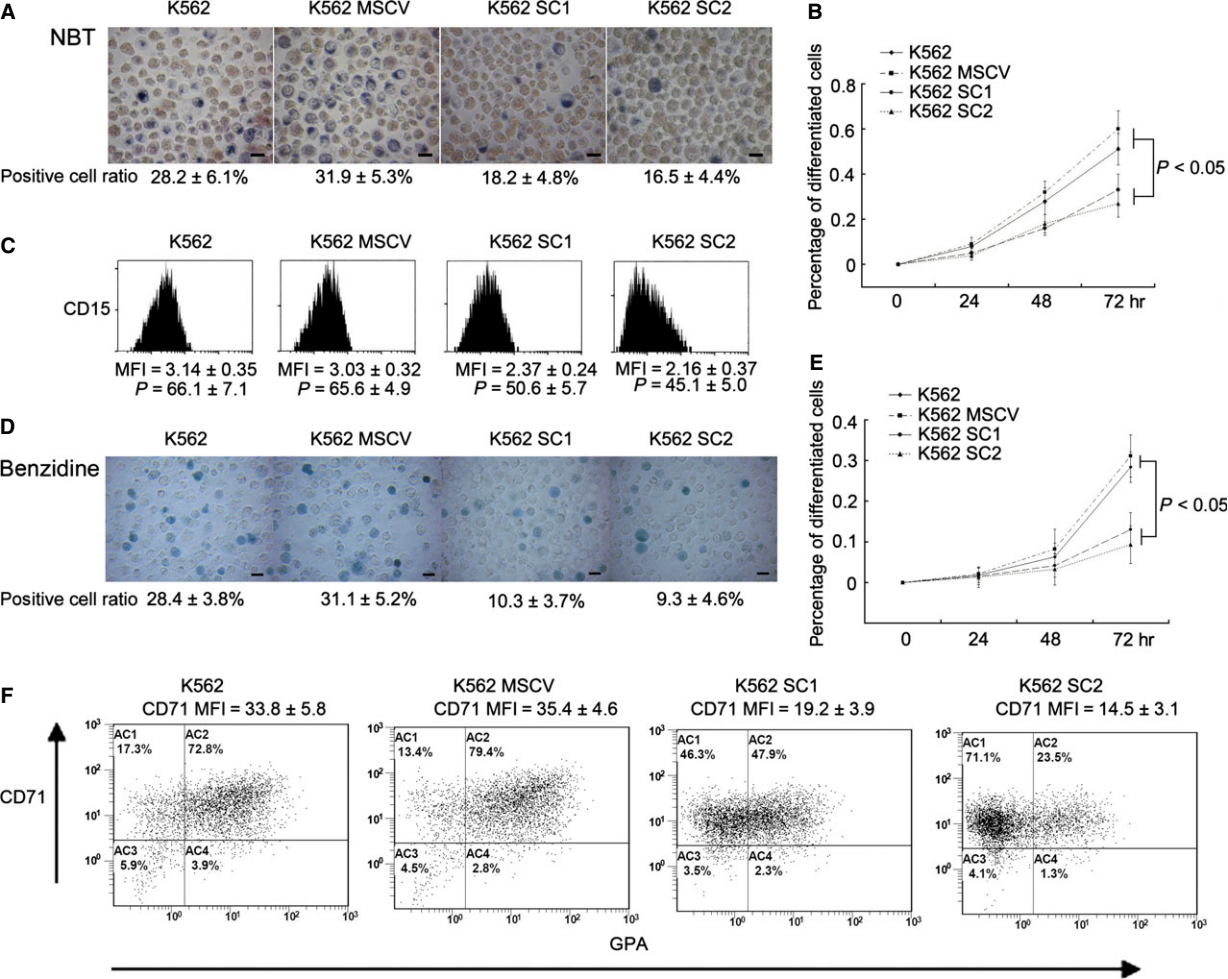
BMI1 inhibits 12-O-tetradecanoyl phorbol-13-acetate (TPA) and histone deacetylase inhibitor sodium butyrate (NaB) induced differentiation in K562 (A) Nitroblue tetrazolium (NBT) assay for cell differentiation induced by 20 nM TPA for 48 hrs. M ± D is the percentage of NBT-positive blue cells, *n* = 3. Bar stands for 10 μm. (B) The differentiation curve of TPA induced differentiation. (C) The mean fluorescence intensity and percentage of CD15 +  cells detected by flow cytometry (FCM) after 20 nM TPA treatment for 72 hrs. Isotype primary conjugated antibodies were served as a negative control. “P” means percentage. (D) Benzidine assay of erythroid differentiation in K562 induced by 0.5 mM NaB for 72 hrs. M ± D is the percentage of benzidine-positive cells, *n* = 3. Bar stands for 10 μm. (E) The differentiation curve of NaB induced differentiation. (F) The dot plot of the FCM analysis for CD71 and GPA staining after cells induced by 0.5 mM NaB for 72 hrs.

Differentiation block is the major mechanism of MDS and CML progression into AML. To further explore the effect of BMI1 upon cell differentiation, we examined the TPA-induced myeloid differentiation and the NaB-induced erythroid differentiation in K562 which had the multiple linage differentiation ability. Nitroblue tetrazolium reduction assay showed that the percentage of differentiated cells in K562 parental cells was considerably higher than that in *Bmi1*-transfected cell after cells treated by 20 nM TPA for 72 hrs (Fig.3A and B). The mean fluorescence intensity and percentage of CD15 positive cells determined by FCM were lower in *Bmi1*-transfected cell than that in control (Fig.3C). Both the NBT reduction assay and the FCM results indicated that the up-regulation of BMI1 hindered TPA-induced myeloid differentiation in K562. Similar results were found in NaB-induced K562 differentiation. Benzidine staining showed that the *Bmi1*-transfected K562 had a lower erythroid differentiation compared to that of the parental control cells after treatment by 0.5 mM NaB for 72 hrs (Fig.3D and E). Furthermore, FCM also showed that the *Bmi1*-transfected K562 underwent a slower NaB-induced erythroid differentiation by detecting CD71 and GPA (Fig.3F). The mean fluorescence intensity of CD71-positive cells in K562 control was much higher than that in *Bmi1*-transfected K562. The ratio of CD71^+^GPA^+^ cells in control K562 was also much higher than that in *Bmi1*-transfected K562. These results indicated that the overexpression of BMI1 inhibited cell erythroid differentiation induced by histone deacetylases (HDAC) inhibitor NaB, implying a possible influence of BMI1 on the histone modification in K562 cells.

### BMI1 inhibits transcription of *Runx1* and *Pten via* multiple histone modification but without direct binding

**Figure 4:**
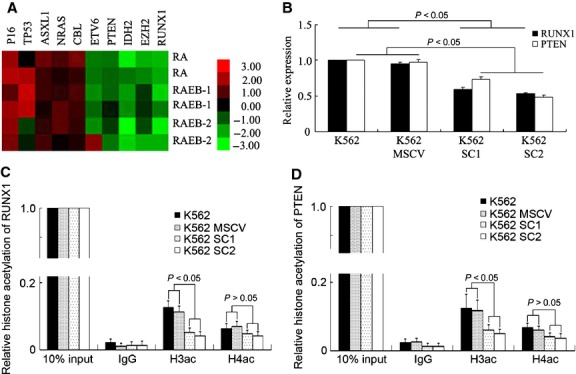
BMI1 indirectly inhibits Runx1 and Pten expression with histone deacetylation. (A) The heat map of poor diagnosis genes in six MDS CD34+ cells of two refractory anaemia (RA), two RA with excess blasts I (RAEB-1) and two RAEB-2 compared to the normal control on the cDNA microarray. (B) The relative transcription of Runx1 and Pten tested by Q-PCR in K562. The transcript level in K562 was set to 1. (C and D) The histone H3 acetylation (H3ac) and histone H4 acetylation (H4ac) at Runx1 and Pten promoter regions tested by chromatin immunoprecipitation (ChIP) and Q-PCR assay in K562, *n* = 3. The histone modification in 10% input DNA was set to 1. IgG was as negative control.

To further verify the correlation of BMI1 with the malignant myeloid progression and a poor prognosis in patients, especially, to explore the molecular mechanism by which the *Bmi1* transfection altered the phenotype of K562 cells, according to Bejar's report, we have firstly analysed the transcription profile of *Runx1, Ezh2, Idh2, Pten, Etv6, Cbl, Nras, Asxl1* and *Tp53* genes in CD34^+^ cells of MDS patients by microarray (Fig.4A) [5]. Among the genes which were down-regulated in all subtypes of MDS patients, *Runx1* and *Pten* were selected and focused on, because the former one is known to be responsible for the regulation of early haematopoietic differentiation, while the latter gene known as a tumour suppressor played pivotal role in distinguishing the leukaemic stem cell from the normal haematopoietic stem cell. Congruously, the transcript levels of both these genes were significantly suppressed in *Bmi1*-transfected K562 cells detected by Q-PCR, *P* < 0.05 (Fig.4B). As BMI1, as a member of polycomb group, participates the epigenetic regulation of target gene *via* joining the PRC1, we have performed a series of ChIP assays to further elucidate the epigenetic regulatory effects on transcription of these two genes exerted by BMI1, including histone trimethylation of H3K9 and H3K27, histone acetylation of H3 and H4. In addition, the interaction of EZH2, a componant of PRC2, with BMI1 in the transfected K562 cells was also studied. The results indicated that the trimethylation of histone H3K27 (H3K27me3) in chromatin correlated with *Runx1* and *Pten* promoter reduced in *Bmi1*-transfected K562 cells. On the other hand, EZH2, which catalyses H3K27me3, was down-regulated in MDS CD34^+^ cells according to MDS CD34^+^ cell microarray and was suppressed in *Bmi1*-transfected K562 cells verified by western blotting. EZH2 suppressed by enforced BMI1 levels was probably responsible for the reduction in H3K27m3 for *Runx1* and *Pten* promoter regions (Data S1).

Meanwhile, the histone H3 acetylation in nucleosomes at *Runx1* and *Pten* promoter regions was down-regulated in *Bmi1-*transfected K562 cells validated by ChIP and Q-PCR, *P* < 0.05 (Fig.4C and D), which was consistent with the results that BMI1 counteracted the differentiation induced by HDAC inhibitor NaB. However, ChIP-PCR results failed to find evidence for the direct binding of BMI1 to the promoter regions of *Runx1* and *Pten*. The ChIP-PCR using antibody against ubiquitin-H2A, a substrate of PRC1, also showed negative results. Therefore, the above epigenetic alterations relevant to suppressing the transcription of *Runx1* and *Pten* must be an indirect effect of BMI1 in K562 cells.

### BMI1 directly suppressed the transcription of *Zmym3* and enhances expression of *c-fos* by modified histone acetylation

**Figure 5:**
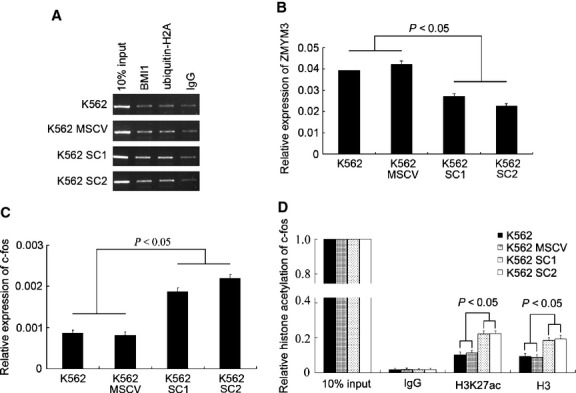
BMI1 directly suppresses Zmym3 and activates c-fos pathway. (A) Electrophoresis strips of Zmym3 promoter region in BMI1 and H2A with ubiquitination ChIP-PCR products. (B) The relative transcript level of Zmym3 in Bmi1-transfected K562 cells. The expression level in K562 was set to 1. (C) The relative transcript expression of c-fos in Bmi1-transfected K562. The expression level in K562 was set to 1. (D) The relative histone H3K27 acetylation (H3K27ac) and H3 acetylation (H3ac) level of c-fos promoter region, *n* = 3. The histone modification in 10% input DNA was set to 1. IgG was as negative control.

To seek the possible direct target gene of BMI1, we have performed ChIP sequencing on the K562 and SKM-1 cells. A list of several dozens of genes with various functions could be obtained and judged to be directly bound by BMI1. Among them, *Zmym3*, which translates a component of histone deacetylase-containing multiprotein complexes, was confirmed to be the direct target of BMI1. Furthermore, ChIP-PCR assay using antibody against ubiquitin-H2A, a substrate of PRC1, also indicated that *Zmym3* gene promoter region could be amplified from the precipitations in K562, U937 and SKM-1. In comparison with the parental K562 cells and K562 cells transfected with MSCV vector, the amplified product of *Zmym3* promoter fragment increased parallelly in both ChIP assays using antibody against BMI1 or against ubiquitin-H2A in the *Bmi1*-transfected K562 cells (Fig.5A). Meanwhile, the transcription of *Zmym3* was markedly reduced, *P* < 0.05 (Fig.5B). To further confirm the effect of BMI1 on the expression of ZMYM3, we looked at the transcription of *c-fos,* one of the ZMYM3 downstream target genes. As being expected, BMI1 increased the transcription of *c-fos*, in company with a higher level of histone H3 and H3K27 acetylation detected by ChIP and Q-PCR, *P* < 0.05 (Fig.5C and D). Taken together, these results indicated that BMI1 suppressed ZMYM3 by direct binding to *Zmym3* promoter region and enhanced the downstream *c-fos* pathway in K562 cells by modifying histone acetylation (Tables3).

**Table 1:** Character of MDS patients

Case	Sex	Age	HB(g/l)	WB(^*^10^9^/l)	PLT(^*^10^9^/l)	Cytogenetic	BLAST^a^	IPSS
1^b^	M	60	74	3.6	15	Complex	3%	1.5
2^b^	M	52	87	3.4	14	-7	2.5%	1
3	M	63	83	3.1	42	Normal	1%	0.5
4^b^	M	70	42	2.1	43	Normal	0.5%	0.5
5	F	69	70	3.8	26	Normal	1%	0.5
6	M	64	76	1.6	79	t(4,21)	0	1
7^b^	M	33	53	3.9	18	Normal	3.5%	0.5
8	F	84	57	4.6	90	Normal	4.5%	0.5
9	M	52	52	1.9	62	Normal	3%	0.5
10	M	65	94	11	77	Complex	3%	1.5
11^b^	M	77	111	8.7	15	Normal	2%	0
12	F	71	57	2.2	45	Normal	1%	0.5
13	F	52	50	1.5	19	Normal	0.5%	0.5
14	M	72	83	3.7	122	Normal	0%	0
15	F	60	78	2.7	78	Normal	0.5%	0.5
16^b^	M	69	58	5.4	458	5q-,20q-	0%	0
17	M	56	63	2.4	56	Normal	0.5%	0.5
18	M	47	71	3.1	68	20q-,+8	0.5%	1
19	M	76	68	2.4	209	Normal	1%	0
20	F	58	54	2.2	74	5q-	1%	0.5
21	M	50	86	2.8	26	Normal	3%	0.5
22	M	52	54	1.6	78	Complex	1%	1.5
23	M	62	75	5.2	18	Normal	0.5%	0.5
24^b^	M	53	78	2.9	116	Normal	0.5%	0
25	M	60	49	11	31	Normal	0.5%	0.5
26	M	17	77	4.7	10	Normal	0	0.5
27	M	66	43	4.4	24	20q-	2%	0.5
28	M	43	121	5.9	72	Normal	0	0
29^b^	M	29	64	6.4	36	Complex	1.5%	1.5
30	M	39	95	2.6	54	Complex	1.5%	1.5
31^b^	M	52	89	21	67	Normal	8.5%	1
32	F	37	58	3.2	44	Normal	6.5%	1
33^b^	F	54	50	2.4	4	Normal	10%	2
34^b^	F	46	78	5.7	810	Normal	8.5%	0.5
35^b^	F	74	62	2	119	Normal	10%	1.5
36^b^	M	39	93	2.8	31	Normal	10%	2
37	M	41	111	3.6	16	Normal	11.5%	1.5
38^b^	M	67	139	3.4	32	Normal	11%	1.5
39^b^	M	64	67	4.7	348	Normal	15.5%	1.5
40	M	51	64	1.4	20	Normal	20%	2.5
41	M	44	40	1.7	24	Complex	9.5%	2
42	M	53	56	1.6	44	Complex	10.5%	3
43	F	64	64	2	88	5q-	16.5%	2
44	M	37	66	6	10	Normal	10%	2
45	F	21	48	4	27	Normal	6.5%	1
46	M	50	56	2.2	19	20q-	7%	1
47	F	77	91	7.2	117	Complex	10%	2.5
48^b^	M	70	104	2.6	18	t(1,1),20q-	20%	2.5
49^b^	F	55	59	1.7	33	+11	7%	1

MDS: myelodysplastic syndromes

HB: haemoglobin

WB: white blood cells

PLT: platelets

M means male

F means female

IPSS: international prognostic scoring system score

“a” blast was calculated in 200 bone marrow karyocytes

“b” CD34+ cells of bone marrow were collected (data in Fig.1C)

“Complex” means more than two abnormities.

**Table 2:** Character of CML patients

Case	Sex	Age	Diagnosis	Cytogenetic
1	M	38	CML	t(9;22),i(17q)
2	F	48	CML	t(9;22)
3	M	47	CML	t(9;22), -Y
4	M	37	CML	Complex
5	F	33	CML	t(9;22)
6	M	46	CML	t(9;22)
7	M	43	CML	t(9;22)
8	F	56	CML	t(9;22),der(15)t(1;15)
9	M	27	CML	t(9;22)
10	M	41	CML	Complex
11	F	62	CML	t(9;22)
12	M	51	CML	t(9;22)
13	F	54	CML	t(9;22)
14	M	28	CML	t(9;22)
15	F	43	CML	Complex
16	M	56	CML	Complex
17	F	60	CML	t(9;22)
18	F	65	CML	t(9;22)
19	M	27	CML	Complex
20	F	40	CML	t(9;22)
21	M	50	CML	t(9;22)
22	F	55	CML	inv(9),t(9;22)
23	F	58	CML	t(9;22)
24	M	47	CML	t(9;22)
25	M	41	CML	t(9;22)
26	M	53	CML	Complex
27	M	24	CML-BP	Complex
28	F	48	CML-BP	Complex
29	F	65	CML-BP	Complex
30	M	28	CML-BP	Complex
31	M	50	CML-BP	Complex
32	M	40	CML-BP	Complex
33	F	47	CML-BP	t(9;22)
34	M	35	CML-BP	t(9;22)
35	M	53	CML-BP	Complex
36	F	34	CML-BP	Complex
37	M	57	CML-BP	t(9;22)
38	M	40	CML-BP	Complex
39	M	46	CML-BP	Complex
40	M	40	CML-BP	t(9;22)
41	M	26	CML-BP	t(9;22)
42	M	47	CML-BP	Complex
43	M	52	CML-BP	Complex
44	F	46	CML-BP	t(9;22)
45	M	30	CML-BP	t(9;22)
46	M	36	CML-BP	t(9;22), +8
47	M	36	CML-BP	Complex
48	F	17	CML-BP	t(9;22)

CML: chronic myeloid leukaemia.

CML-BP: chronic myeloid leukaemia in blast phase.

“complex” means more than two abnormities.

**Table 3:** Cytogenetic of MDS patients in CD34+ microarray (Fig.4A)

Case	Sex	Age	Cytogenetic
RA-1	F	53	Complex
RA-2	M	40	Complex
RAEB1-1	M	48	der(6), +8
RAEB1-2	F	40	5q-
RAEB2-1	M	34	dup(1),tandem
RAEB2-2	F	54	+8

RA: refractory anaemia.

RAEB: refractory anaemia with excess blasts.

“Complex” means more than two abnormities.

## Discussion

A considerable portion of MDS patients, even being firstly diagnosed as RA or RAEB, will eventually undergo malignant myeloid progression into AML. The factors led to this transformation of MDS patients are not well understood. However, a clinical study on a relative large cohort of MDS patients reported that the BMI1 was associated with this process and predicted a poor prognosis of the MDS patients. Similarly, most of the patients with CML-CP also have the process of malignant myeloid progression from CP to BP during the clinical course. Despite of a different pathogenic background for patients with MDS and CML-CP, such as occurrence of t(9;22)/BCR-ABL in CML and variety of chromosomal abnormalities in MDS, common cell phenotypic changes are shared by the aberrant myeloid progenitor cell clones in both MDS and CML during the advanced stage of malignant myeloid progression or so-called secondary AML (sAML). The main common phenotypic alterations are that the abnormal cell clones, which previously somehow retain differentiation capacity, lose this capacity and become differentiation blocked. Meanwhile, its relatively normal proliferation rate, which allows a delicate balance maintained in the bone marrow between the aberrant and normal clones, rapidly elevates to become uncontrollable. Focusing at this clinical stage, interestingly, we and others found that a markedly increasing transcript level of *Bmi1* in the CD34^+^ bone marrow cells or BMMCs was correlated with the transformation of MDS into AML, as well as with the evolution of CML from CP to BP. Furthermore, the *Bmi1* transcription in the CD34^+^ cells of MDS-derived AML or in the BMMCs of CML-BP was even much higher than that of the respective dAML, indicating that the high level of BMI was not because of high ratio of blast cells. These findings implied that, in addition to the respective pathogenic mechanism of these two haematological diseases, a possible common mechanism may govern the terminal stage, namely the malignant myeloid progression, and wherein BMI1 seemed to play a pivotal role.

BMI1 has been known as the core member of polycomb repressive complex 1, affects to maintain the self-renewal capacity and undifferentiated status of stem cells. It collaborated with *Hox* gene suppressed cellular senescence *via* inhibiting P16^INK4/ARF^ to preserve the stemness of normal haematopoietic stem cells. During leukaemogeneis, the leukaemic fusion genes, such as MLL-AF9 or PML/RARα and AML1/ETO, need BMI1 to suppress the oncogene-induced senescence. It is essential for faithfully epigenetic reprogramming to establish a leukaemic stem cell-specific gene expression profile to ensure a fully conversion of normal myeloid progenitor cells into leukaemic stem cells [27,34]. Thus, one can easily reason that the aberrant BMI1 levels inevitably disturb the gene expression profile of the haematopoietic stem cell, which may act sequentially or synergically with the existing genetic changes to promote malignant myeloid transformation in both MDS and CML. Besides, the significant higher transcription of *Bmi1* in AML evolved from MDS or CML than that in dAML seemed suggest a more critical role in the gradual process of malignant myeloid transformation.

To elucidate the action and its mechanism of BMI1, the biological features were carefully examined after the transfection and overexpression of BMI1 in K562 cells, because the well-characterized cells represented the typical CML cell with a multilineage differentiation potential. The BMI1 clearly hindered the mutilineage differentiation of K562 cells induced by respective inducers. The larger size of replated colonies implied a stronger self-renewal capacity of the transfected cells although the cell growth rate did not change much. Most impressively, the *Bmi1*-transfected cells became more resistant to the ATO-induced apoptosis. Moreover, the TPA-induced and HDAC inhibiter NaB-induced differentiation were also inhibited in these transfected cells, suggesting that BMI1 probably played a role in histone acetylation modification.

Recently, the genetic lesions associated with sAML were studied by high-resolution SNP genotyping and loss of heterogeneity in comparison with dAML. The leukaemogenic mechanism must substantially differ between sAML and dAML [35,36]. These sAML mainly refer to those who arise from MDS or MPN. Mutations in *Tp53*, 9pUPD and del17q have been identified as independent adverse prognostic biomarkers to be significantly associated with sAML. In our present study, the AML evolved from MDS or CML was also viewed as sAML. In this sense, we identified the elevated expression of BMI1 as a common feature of sAML in comparison with dAML.

The mechanisms of MDS development are heterogeneous. *Tp53* or *Etv6* deletion was frequently happened in high risk MDS patients [37,38]. Thus, MDS patients probably have a distinct expression level of TP53 and ETV6 in accordance with our microarray results. As *P16* and *Tp53* are homozygously deleted in K562 leukaemia cell line, BMI1 must affect *via* some important functioning pathways other than direct binding to *Ink4a/Arf* locus in K562. To identify some possible target genes of BMI1 which may be closely related to the altered cell phenotypes, *Runx1* and *Pten* drew our attention. A number of studies have reported that MDS with loss-of-function *Runx1* mutation has a poorer prognosis [6,31,35]. Chronic myeloid leukaemia also has a high rate of *Runx1* mutation in BP [16]. Meanwhile, *Pten*, as a tumour suppresser gene and contributing to leukaemia development, inhibits BMI1 promoting stem cell self-renewal and tumourigenesis, indicating that PTEN rescues the phenotypes by the overexpression of BMI1 [39–41]. The loss-of-function mutation or down-regulation of these two genes both lead to their deficient function and result in the malignant progression of the clonal myeloid cells. In the meantime, our pilot study on the gene expression profile of the MDS CD34^+^ cells by microarray showed that the transcriptions of *Runx1* and *Pten* were down-regulated in MDS CD34^+^ cells compared to the non-MDS normal controls. Accordingly, we confirmed that BMI1 inhibits the transcription of *Runx1* and *Pten* in K562 and U937.

The alterations of histone methylation, including the gene activating markers H3K4m3, H3K36m and H3K79m and the gene repressive markers H3K9m and H3K27m3, as one of the major epigenetic mechanism is presently studied more frequently in MDS, while histone acetylation which should facilitate the gene transcription is less mentioned. Theoretically, the methylation and acetylation modification at a specific lysine residue of a particular histone molecule should be mutually exclusive. However, chromatin-modifying enzymes with different catalytic activities usually function *via* forming multiple protein transcriptional regulatory complexes containing REST and HDAC1/2 *etc*. [42]. Thus, the overall influence of these histone modifications on the transcription of certain gene depends on the collective consequence of individual histone modifiers recruited to the gene promote location. In our present study, apart from the relatively subtle change in H3K27m3 associated with *Runx1* and *Pten* in *Bmi1*-transfected K562 cells, more interestingly, we found a marked histone deacetylation, especially for H3 associated with these two genes in the *Bmi1*-transfected cells. Although the effect of reduced H3K27m3 and the down-regulated H3 acetylation on *Runx1* and *Pten* seemed contradictory, the overall consequence was the decrease in their expression. More specifically, the increasing acetylation of H3 and H3K27 associated with up-regulation of *c-fos* were also detected in these cells. These results emphasize that BMI1 probably plays a pivotal role of histone acetylation or deacetylation in transcriptional regulation.

HDAC inhibitor induces cell senescence by increasing histone acetylation of a number of target genes and down-regulation of BMI1 [43]. Thus, the overexpression of BMI1, collaborated with genes histone deacetylation such as *Runx1* and *Pten*, is able to resist cell senescence and oppose the differentiation induced by HDAC inhibitor. BMI1 is usually recruited to target chromosomal site simultaneously with decreased lysine acetylation [44]. However, the mechanism remains still unclear; probably, the PRC1 complex “protects” the target DNA sequences from histone H3 acetylation. Our ChIP-PCR results seemed indicate that neither BMI1 nor ubiquitin-H2A directly bound to the promoter region of *Runx1* or *Pten* in leukaemic cells. So the influence exerted by BMI1 upon these two genes must be through some indirect interactions. All together, the BMI1-induced repression of *Runx1* and *Pten* may convey a more malignant phenotype in those abnormal haematopoietic stem/progenitor clones, leading to malignant myeloid progression in MDS or CML-CP.

To further seek the target gene and possible downstream pathway of BMI1, especially in the K562 cells lacking of *P16* and *Tp53* genes, by screening the ChIP-seq and ChIP-PCR data, we found that BMI1 suppressed the transcription of *Zmym3* by directly binding its promoter region, thus opened a novel pathway circumventing the *P16* or *Tp53*-mediated pathways. ZMYM3, the candidate for X-linked mental retardation also known as ZNF261, XFIM or DXS6673E, is a component of histone deacetylase-containing complex BHC formed by HDAC1/2, BHC-110, CoREST, BRAF35, BHC-80, TFII-I, KIAA0182, ZMYM2 and ZMYM3, and is required for the maintenance of long-term repression of neuronal-specific genes [45]. The ZMYM3-containing multiprotein complex was specifically recruited to the promoter of *c-fos* by TFII-I and thus suppressed the expression of numerous target genes including *c-fos* [46,47]. Accordingly, as a target gene of ZMYM3, we further demonstrated an elevated level of *c-fos* expression as the consequence of suppression of *Zmym3* associated with an increasing acetylation at H3 in the *Bmi1*-transfected K562. ZMYM3 containing histone deacetylase complex deacetylates the whole H3 acetylation of *c-fos* and is not a specific deacetylase for H3K27 acetylation. For the loss of PRC2 activity results in a global increase in H3K27 acetylation, we further verified that *c-fos* had an increasing H3K27 acetylation level in promoter region when EZH2 was down-regulated in *Bmi1*-transfected K562 [48]. In K562, *c-fos i*s proved to counteract with *c-Jun* in Fas/FasL pathway, which mediates cell death and apoptosis [49,50]. As a protooncogene with oncogenic function, the up-regulation of *c-fos* may contribute to the malignant myeloid progression of MDS into AML [51]. Thus, the activation of *c-fos via* a HDAC1/2 containing multiprotein BHC complex may provide an alternative pathway for BMI1 to render more malignant phenotype in the myeloid cell clones, especially in the *P16* deleted K562 cells or *P16* mutant MDS cells. *Zmym3* may act as another important target gene of BMI1, especially in P16 deficient clonoal myeloid cells.

In summary, we have demonstrated that BMI1, as an oncoprotein, block the differentiation and strengthen the viability of the clonal myeloid leukaemic cells by reprogramming the histone acetylation profile for variety of downstream genes, for example, deacetylating H3 for *Runx1* and *Pten via* indirect action and enhancing H3 acetylation for *c-fos via* direct binding to promoter of *Zmym3* and suppressing the function of ZMYM3-containing multiprotein complex. BMI1 is useful as a biomarker for predicting malignant myeloid progression in a subset of either MDS or CML-CP by epigenetically reprogramming the H3 acetylation profile which also may contribute to the poor prognosis of these diseases.

## Supporting Information

**Data S1** Primers for PCR. **Figure S1** Negative control of CD15 in paper Fig. 3C. **Figure S2** Negative control of GPA and CD71 in paper Fig. 3F. **Figure S3** PI staining was performed to determine cell cycle and there was no significant alternation in transfected K562 compared to control. **Figure S4** Growth curve was detected by MTT method and there was no significant proliferation alternation in transfected cell compared to control (K562 SC3 was not mentioned in paper). **Figure S5** ChIP-sequencing (Illummina HiSeq 2000) results of BMI1 in K562. **Figure S6** ChIP-sequencing results of BMI1 in SKM-1 cell line (cell line of MDS progressed into AL). BMI1 also binds Zmym3 in SKM-1. **Figure S7** BMI1 functions in U937 similar with that in K562. **Figure S8** (A) Western blot of EZH2 in Bmi1 transfected K562. **Figure S9** Apoptosis ratio of K562 treated by 100 μM imatinib (STI-571, Selleck Chemicals, Houston, TX, USA) for 24 hrs. BMI1 did not increase the resistance to imatinib in K562.
