# Supplementary material for: BMI1 reprogrammes histone acetylation and enhances c-fos pathway via directly binding to Zmym3 in malignant myeloid progression
Source: J Cell Mol Med. 2014 Feb 27;18(6):1004–17. doi: 10.1111/jcmm.12246 (PMC4508141; doi:10.1111/jcmm.12246)
Supplement: Supplementary file 1 [file jcmm0018-1004-sd1.doc]

**Supplemental**

**Official document of the biomedicine research ethical committee of the First affiliate Hospital, Soochow University page1**


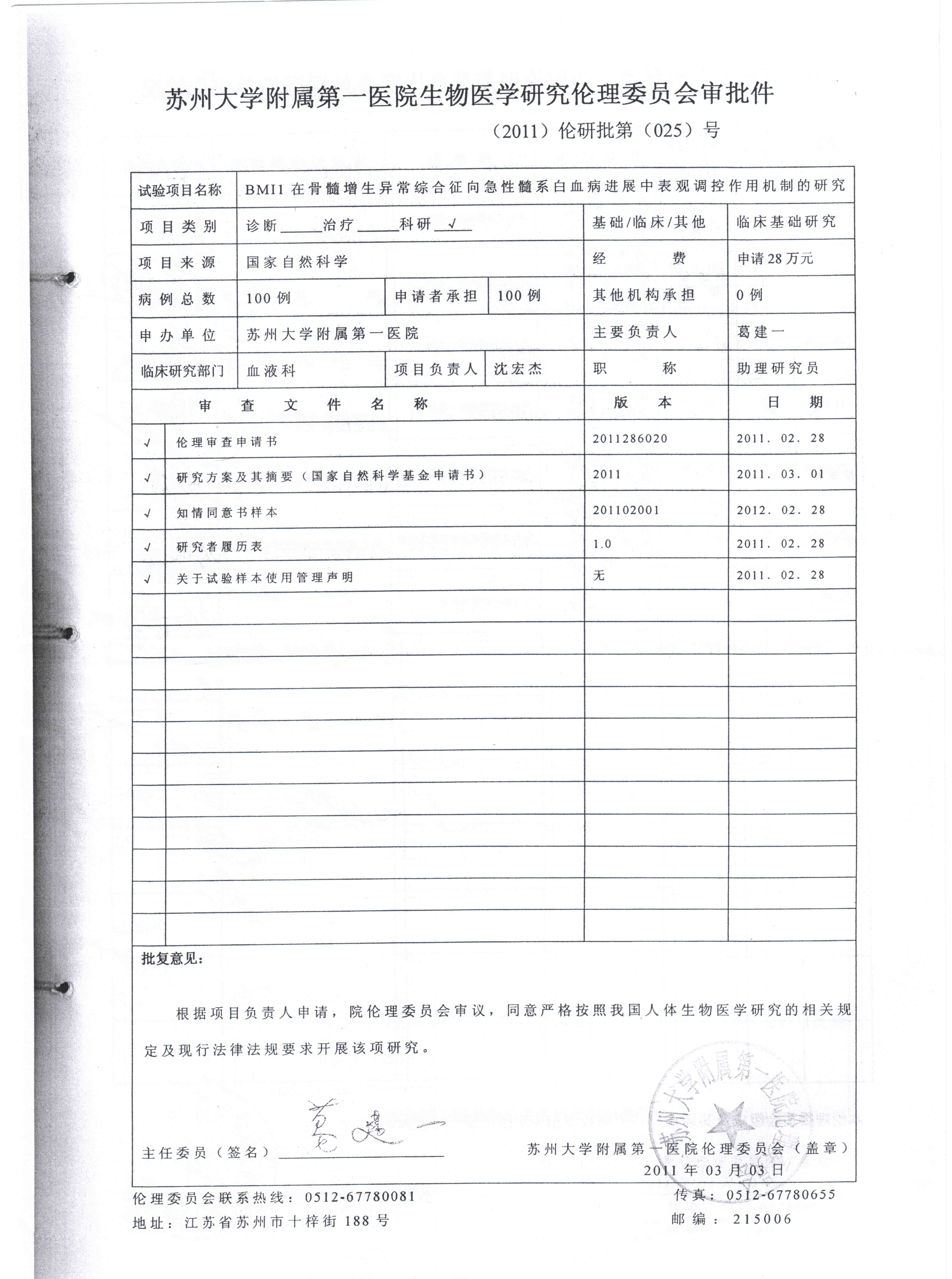


First 6 lines of the up table

| Name | Study on the epigenetic mechanism of BMI1 in MDS transformed AML | | | | | | |
| --- | --- | --- | --- | --- | --- | --- | --- |
| type | diagnosis clinical research √ | | | | | preclinical/clinical/other | preclinical |
| grant support | the National Natural Science Foundation of china | | | | | Fee | ￥250,000 |
| Number of cases | 100 | applicants undertake | | | 100 | Others undertake | 0 |
| organization | the First affiliated Hospital of Soochoow University | | | | | Responsible person | Jianyi ge |
| department | heametoledge | | project leader | Hongjie Shen | | title | assistant Research |

Official document of the biomedicine research ethical committee of the First affiliate Hospital, Soochow University page2


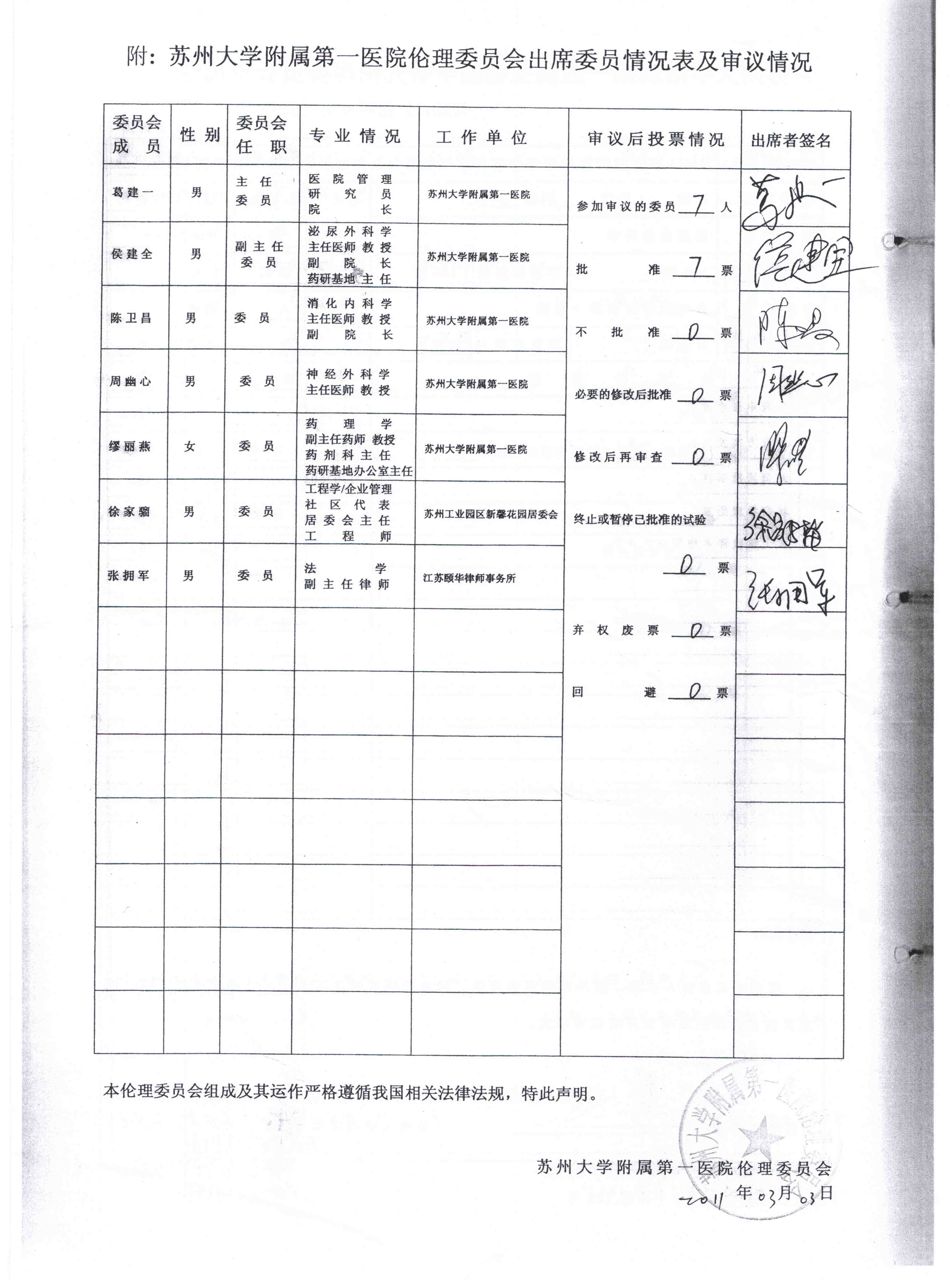


**Menbers of the biomedicine research ethical committee of the First affiliate Hospital, Soochow University**

1. Jianyi ge. chairman. Director of hospital, senior researcher. the First affiliate Hospital of Soochow University.
2. Jianquan Hou. Vice chairman. chief physician of Urology, Professor. the First affiliate Hospital of Soochow University.
3. Weichang chen. Member. chief physician of gastroenterology, Professor. the First affiliate Hospital of Soochow University.
4. Youxin Zhou. Member. chief physician of neurosurgery, Professor. the First affiliate Hospital of Soochow University.
5. Liyan Miao. Member. associate chief pharmacist, Professor. the First affiliate Hospital of Soochow University.
6. Jialiu Xu. Member. chief of residents' committee, community representatives, Engineer. Director of Soochoow industry park.
7. Yongjun Zhang. Member. Lawyer. Yihua lawyers' office of Jiangsu provience, china.

**Written informed consent case 001 in this paper (as example):**


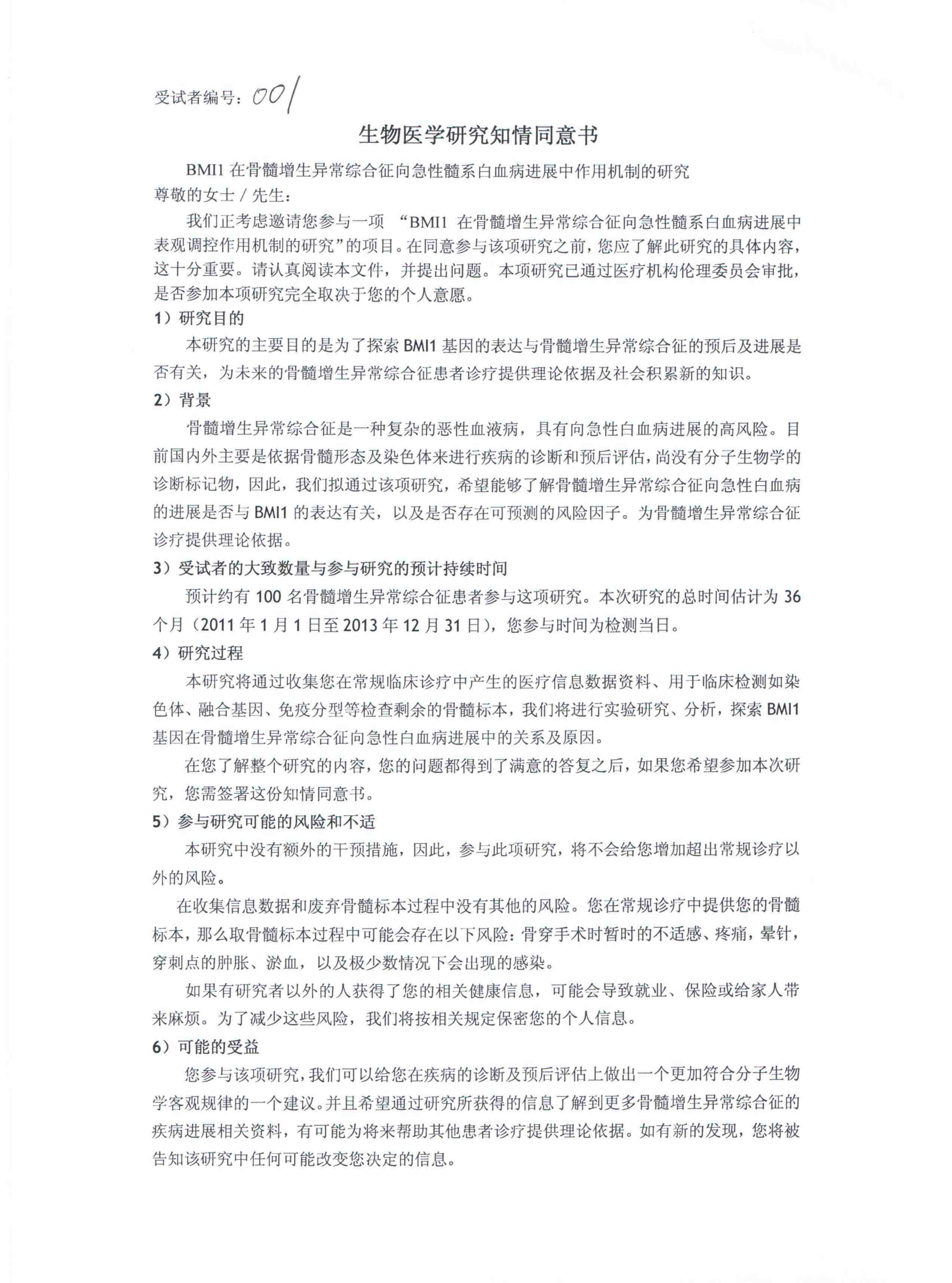

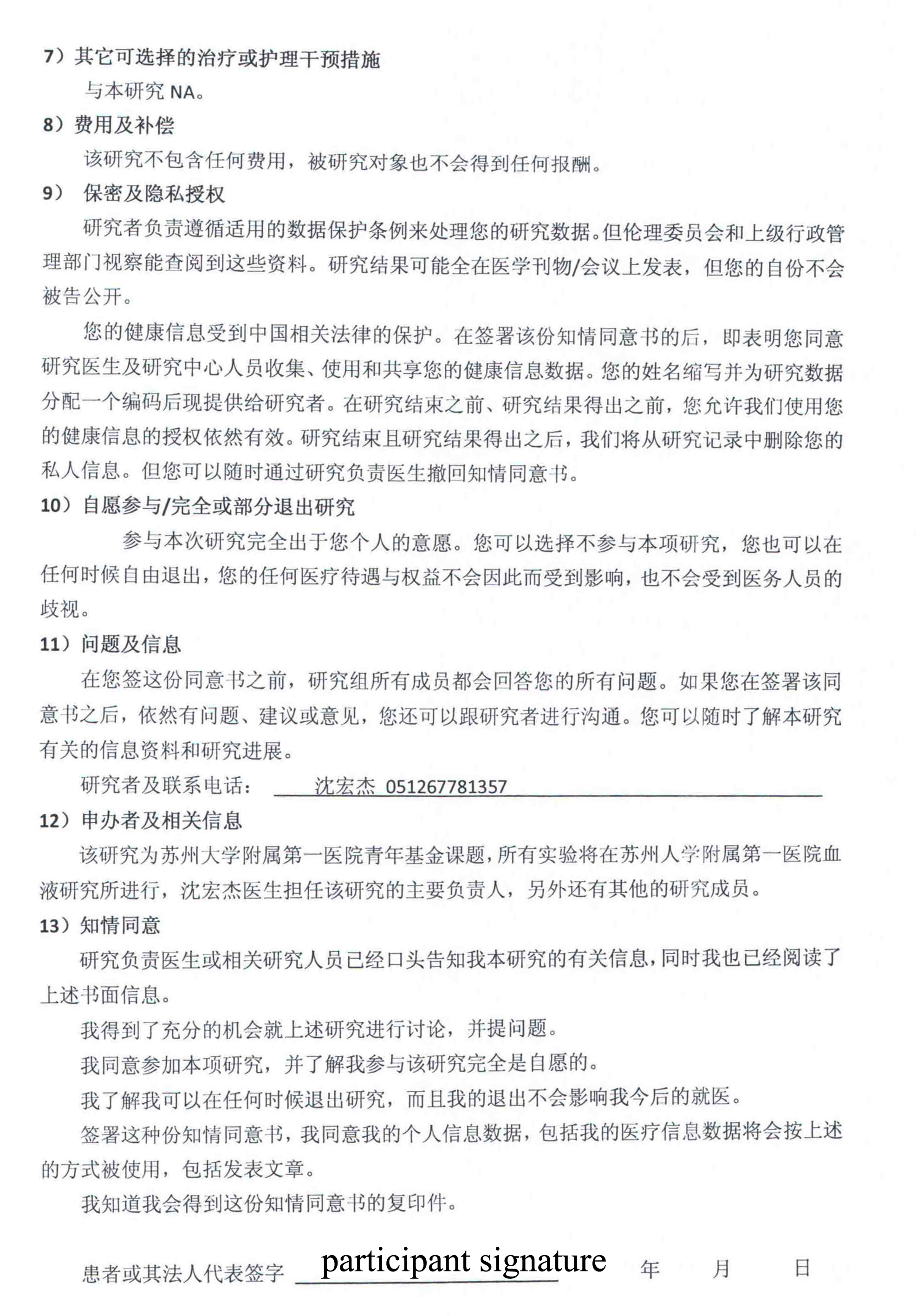


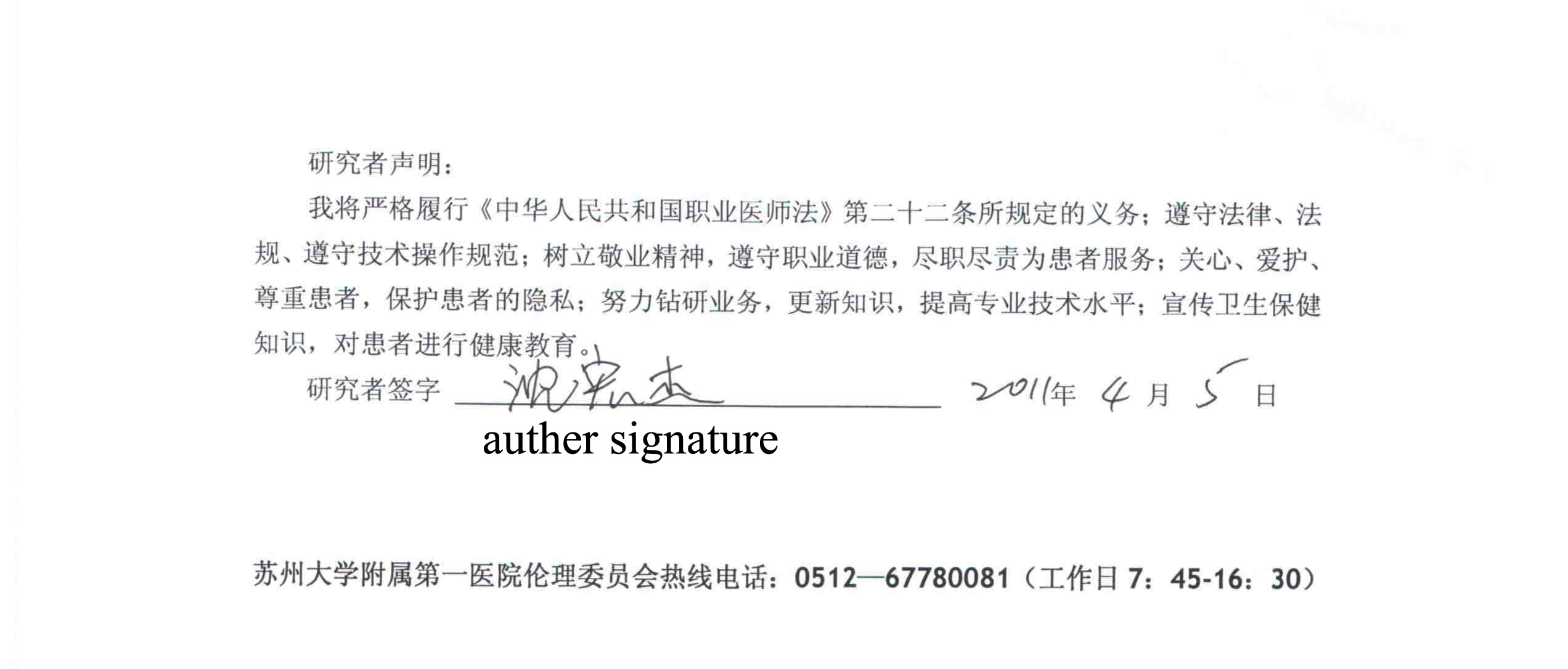


The main procedure of signing informed consent:

Firstly, the participants were told about all the content in the informed consent: the name of the subject, the background of this subject, the process of this subject, the benefits and the possible risk of participants would have, the private of participants would be protected and not mentioned in paper, only the clinical materials and research results could be used and published in paper, and so on. Secondly, if the participants agreed to participate in this study, he or she signed the informed consent. All the processes were under the legal program.

**Primers and probes for Q-PCR**

BMI1：

F:5’-ctgctgaacgacttttaactttcatt-3’; R:5’-cttgataaaaaatcccggaaagag-3’

P:5’-fam-tcttttccgcccgcttcgatcg-tamra-3’

GAPDH：

F: 5’-gctcctcctgttcgacagtca-3’; R: 5’-accttccccatggtgtctga-3’

P: 5’- fam-ccgcatcttcttttgcgtcgcc-tamra -3’

PTEN：

F:5’-tgtggtctgccagctaaaggt-3'; R:5’-gctgagggaactcaaagtacatga-3’

P:5’-fam-atattcctccaattcaggacccacacgac-tamra-3’

SYBR green was used to detect the relative expression of RUNX1,ZMYM3,c-fos.

RUNX1:

F:5’-tgagcccaggcaagatga-3’; R:5’-agcacggagcagaggaag-3

ZMYM3:

F:5’-atgtggtgcggcagtcc-3’; R:5’-gtgtctcgccctttcctct-3’

c-fos:

F: 5’- tttgcctaaccgccacgat -3’; R: 5’- ctgcgggtgagtggtagtaagag -3’

GAPDH (same as above )：

F: 5’-gctcctcctgttcgacagtca-3’; R: 5’-accttccccatggtgtctga-3’

**Promoter region primer**

The sequences of promoter region were obtained from <http://rulai.cshl.edu/cgi-bin/CSHLmpd2/promExtract.pl?species=Human> and predicted from <http://www-bimas.cit.nih.gov/molbio/proscan/>.

RUNX1 promoter primers (NM_001754.4):

F: 5’-gcgtggctgctttcaactttcc-3’, R: 5’-tcttcccttgggtcggtttctg-3’

Pten promoter primers:

F: 5’-ccgactgtggcccgtgtatc-3’, R: 5’-gtggaaagtacggaacggtaggaa-3’

(NM_000314, the promoter region is also accordance with Sheng’s report.

Sheng X, Koul D, Liu JL, Liu TJ, Yung WK. Promoter analysis of tumor suppressor gene PTEN: identification of minimum promoter region. Biochem Biophys Res Commun. 2002;292(2):422-426.)

Zmym3 promoter primers ([NM_005096.3](http://www.ncbi.nlm.nih.gov/nuccore/283837892)):

F:5’-ggggagcaggtgcgtagat-3’; R:5’-tggaaattgcggagtgga-3’

C-fos promoter primers (cited from Endocrinology. 1995;136(7):3046-3053. Role of specific response elements of the c-fos promoter and involvement of intermediate transcription factor(s) in the induction of Sertoli cell differentiation (transferrin promoter activation) by the testicular paracrine factor PModS.)

F:5'-agcagttcccgtcaatcc-3'; R:5'-tgagcatttcgcagttcc-3'


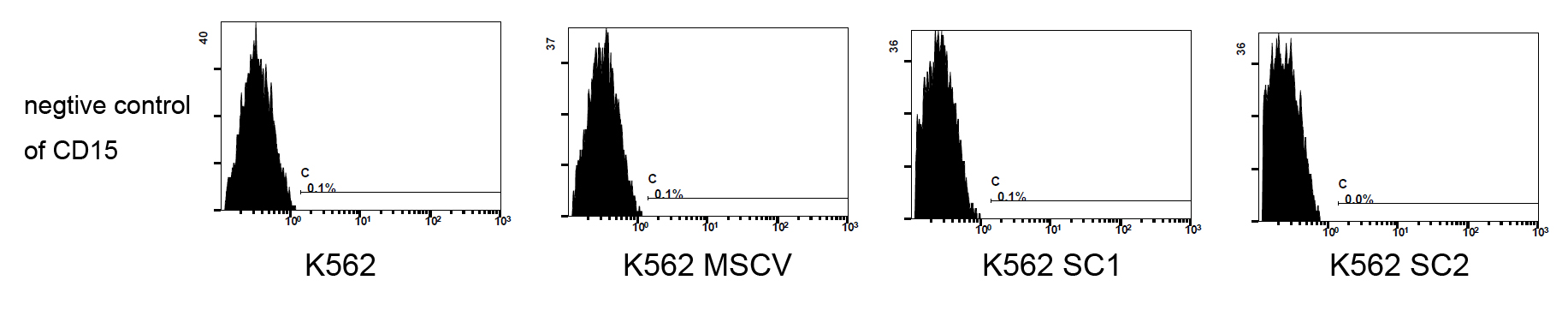


Fig 1 Negtive control of CD15 in paper Fig3 C.

Fig 2 Negtive conrol of GPA and CD71 in paper Fig3 F.

K562 K562 MSCV BMI1 K562 SC1 BMI1 K562 SC2


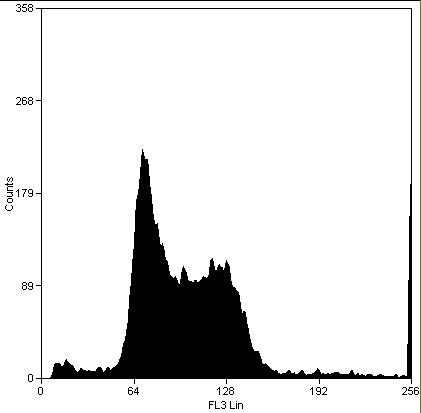

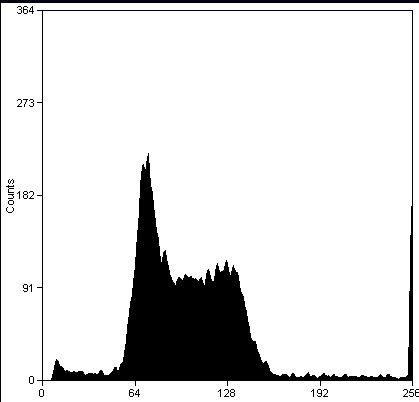

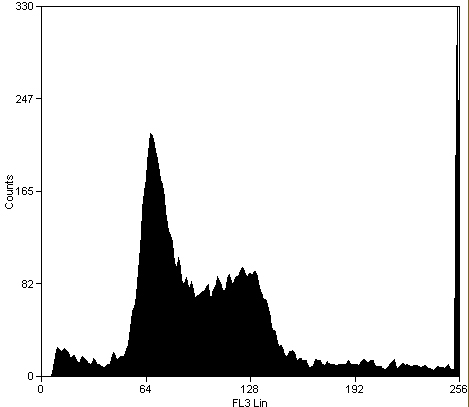

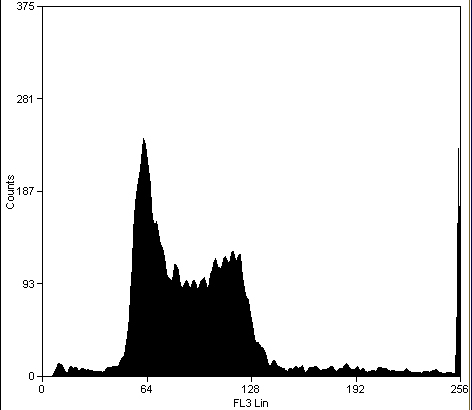


Fig 3 PI staining was performed to determined cell cycle and there was no significant alternation in transfected K562 compared to control


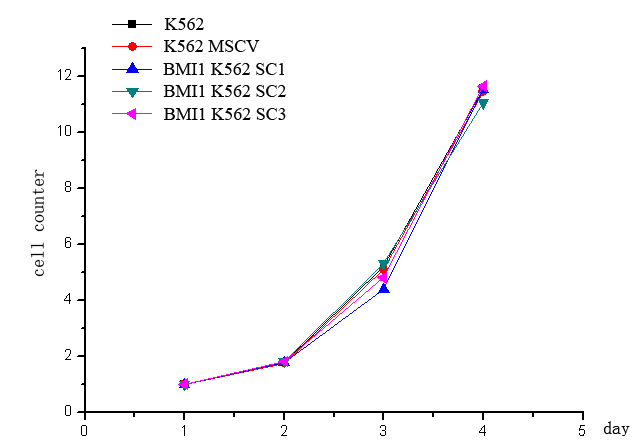


Fig 4 Growth curve was detected by MTT method and there was no significant proliferation alternation in transfected cell compared to control (K562 SC3 was not mentioned in paper)


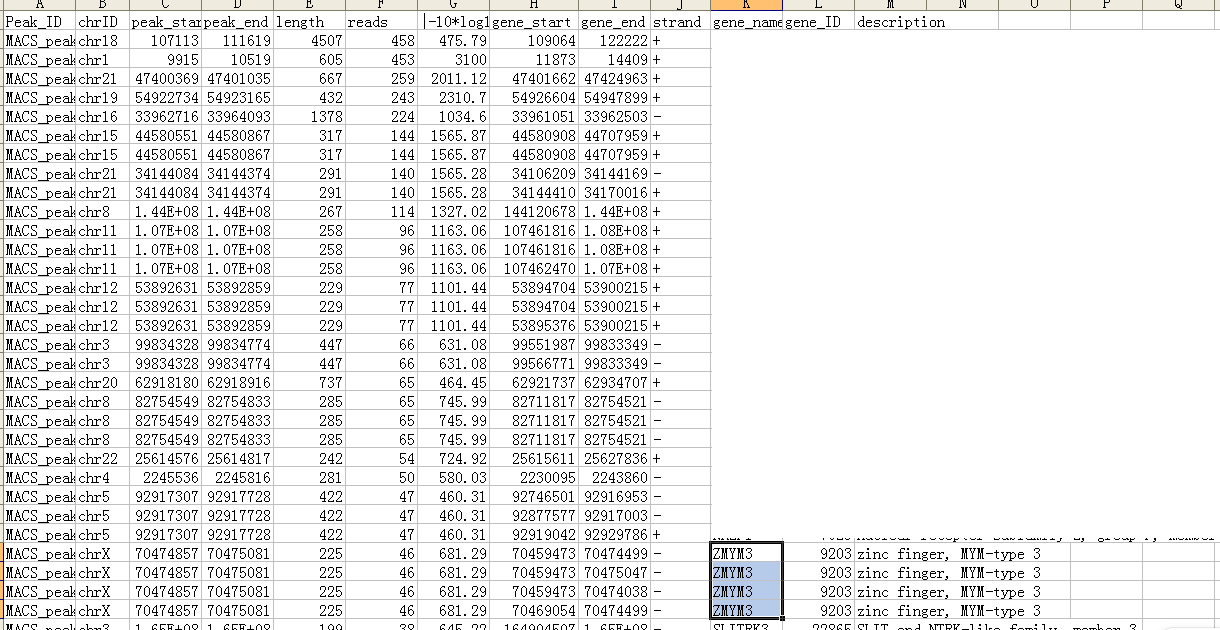


Fig 5 ChIP-sequencing (Illummina HiSeq 2000) results of BMI1 in K562.


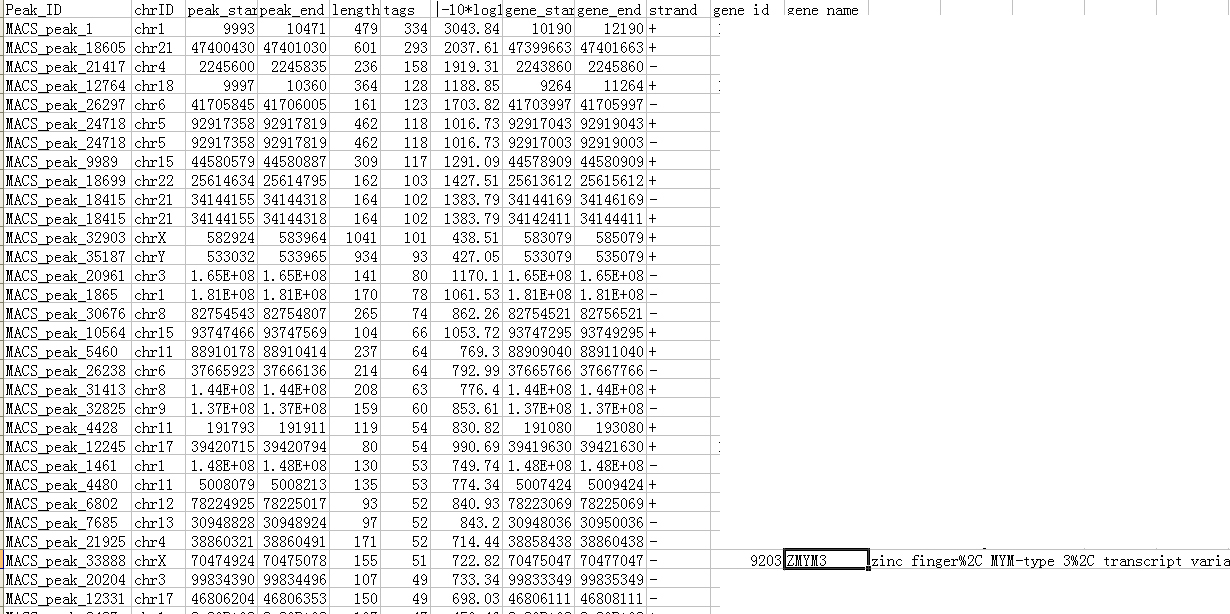


Fig 6 ChIP-sequencing results of BMI1 in SKM1 cell line (cell line of MDS progressed into AL). BMI1 also binds Zmym3 in SKM1.

We also confirmed that BMI1 directly bind the promoter region of Zmym3 in SKM1 verified by ChIP-PCR. Other genes were not verified by ChIP-PCR and did not show in this supplemental.


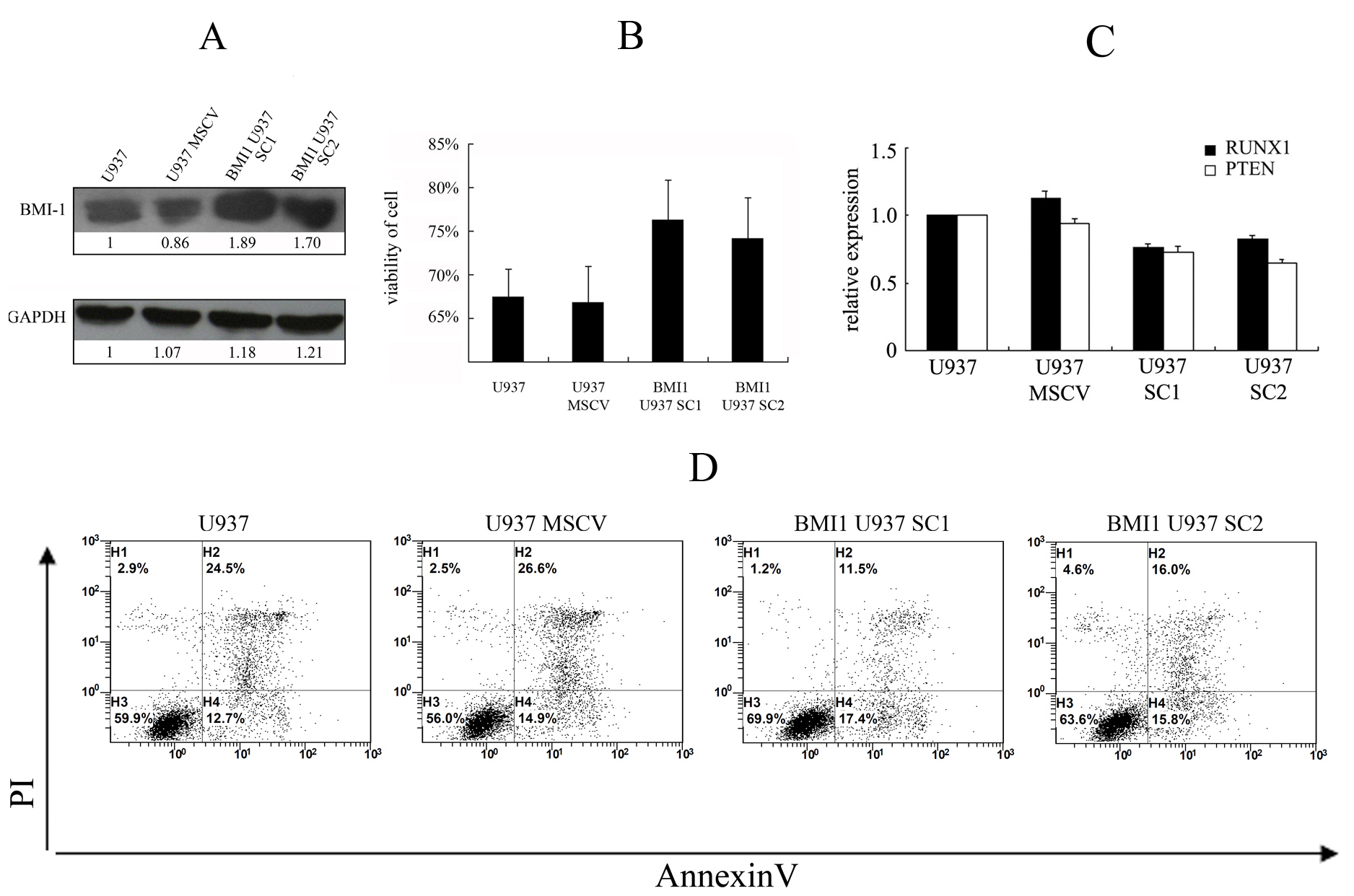


Fig 7 BMI1 functions in U937 similar with that in K562. A) Western blot of BMI1 in Bmi1 transfected U937. B) BMI1 increases U937 viability after cells are cultured without FBS for 72 hours, *p*<0.05 C) BMI1 inhibits Pten and Runx1 transcript expression, *p*<0.05. D) BMI1 counteracts ATO induced apoptosis in U937.

Although BMI1 protein level was not 2-folds more than control (Fig7 A), there was a tendency that BMI1 inhibited Runx1 and Pten expression in U937 (Fig 7A,C). The inhibitions of Runx1 and Pten were not unique in Bmi1 transfected K562. BMI1 also increased U937 cell viability and counteracted apoptosis in U937 (Fig7 B,D). From the apoptosis results of K562 (Fig2 D in paper) and U937 (Fig7 D in this supplemental), BMI1 seemed not to reduce the ratio of early apoptotic cells but mainly slow down the progression of early apoptotic cells into late apoptotic cells. Therefore, BMI1 counteracts ATO induced apoptosis both in K562 and U937.


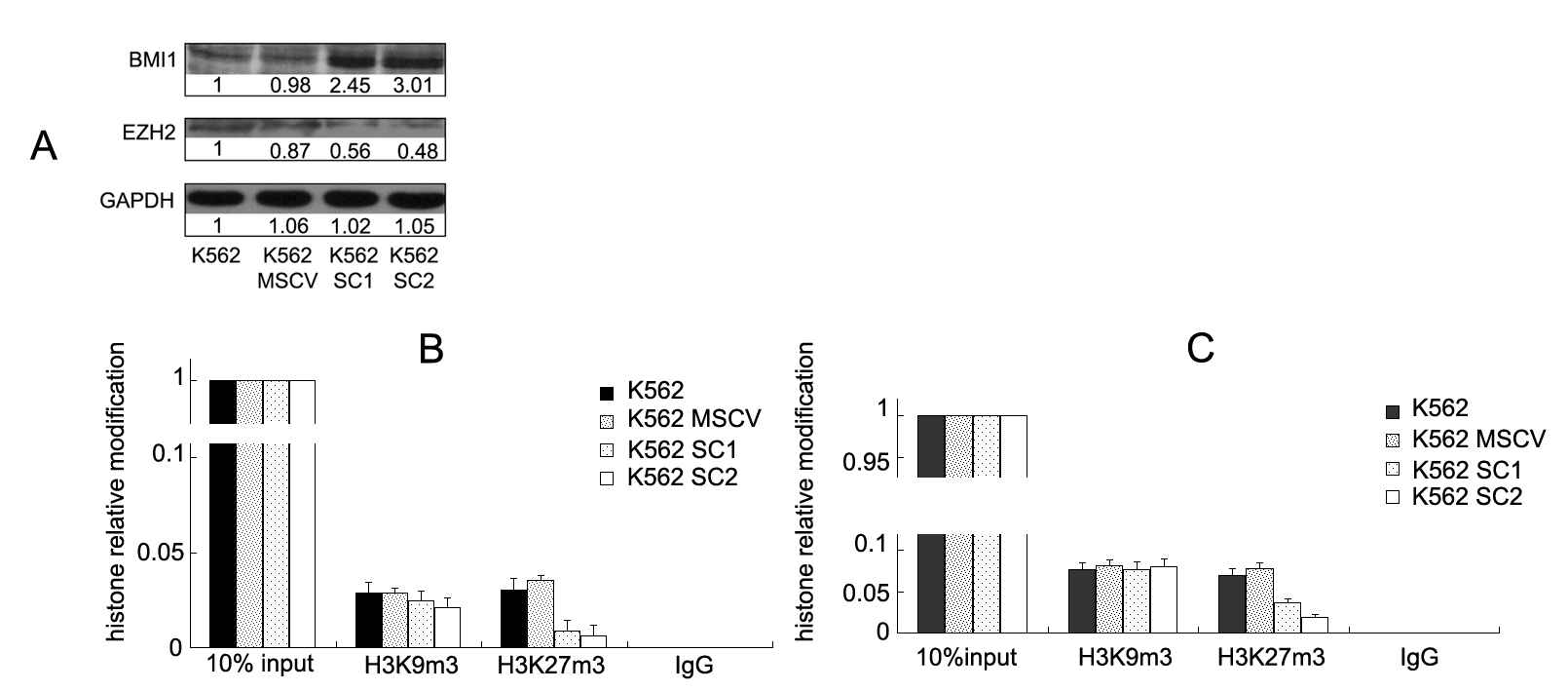


Fig8 A) Western blot of EZH2 in Bmi1 transfected K562. B) The histone relative modification of Runx1 promoter region. The Runx1 promoter H3K27m3 level in Bmi1 transfected K562 was higher than that of control, *p*<0.05. C) The histone relative modification of Pten promoter region. The Pten promoter H3K27m3 level in Bmi1 transfected K562 was higher than that of control, *p*<0.05. 10% input is set to 1.

BMI1 inhibited EZH2 in K562 (Fig8 A).There is no clearly alternation of K3K9m3 but a reduced histone modification of H3k27m3 in Runx1 and Pten promoter region (Fig8 C,D). We also provide that the deficiency of EZH2 leaded to the down-regulation H3K27m3 both in Runx1 and Pten promoter region by EZH2 siRNA. However, the low trimethylation levels of H3K9 and H3K27 indicated that H3K9m3 and H3K27m3 were probably not the main reasons for the down-regulation of Runx1 and Pten in Bmi1 transfected K562.


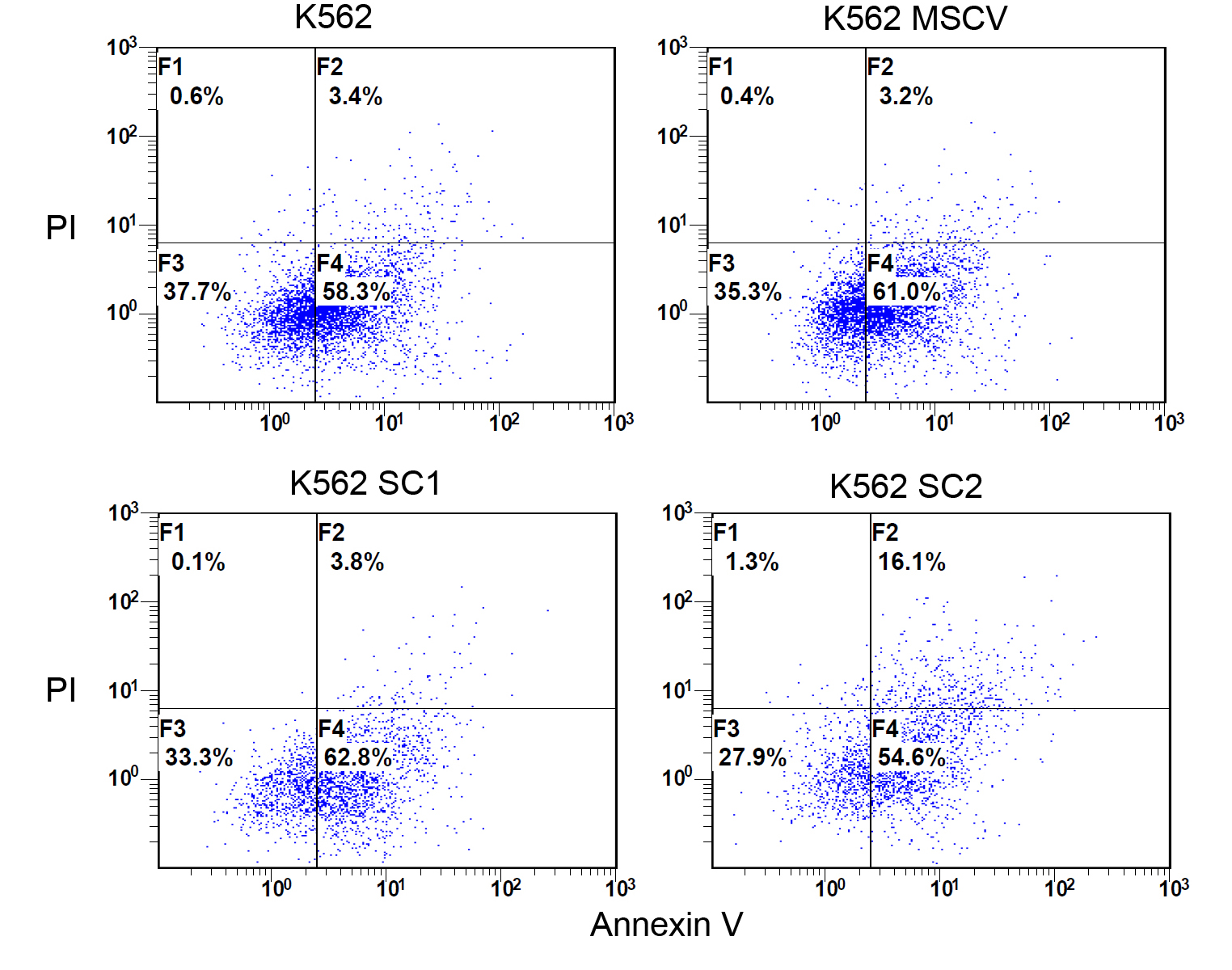


Fig9 Apoptosis ratio of K562 treated by 100μM imatinib (STI-571, selleckchem) for 24 hours. BMI1 did not increase the resistance to imatinib in K562.
